# Retracted: Rosiglitazone Suppresses the Growth and Invasiveness of SGC-7901 Gastric Cancer Cells and Angiogenesis In Vitro via PPAR*γ* Dependent and Independent Mechanisms

**DOI:** 10.1155/2020/9469261

**Published:** 2020-09-01

**Authors:** PPAR Research

*PPAR Research* has retracted the article titled “Rosiglitazone Suppresses the Growth and Invasiveness of SGC-7901 Gastric Cancer Cells and Angiogenesis In Vitro via PPAR*γ* Dependent and Independent Mechanisms” due to figure duplication.

As raised on PubPeer [2], Figure 3C shows several issues:

- For PPAR*γ*, lanes 1, 2, and 3 appear similar
- For PPAR*γ*, lanes 6, 7, 9, and 11 all appear similar
- For PPAR*γ*, lanes 8 and 10 appear similar
- For MMP-2, lanes 1 and 7 appear similar; likewise lanes 3 and 8
- For VEGF, lanes 8 and 11 appear similar
- For *β*-actin, lanes 1, 2 appear similar to lanes 7, 8
- For *β*-actin, lanes 5, 9, and 11 appear similar

At revision, the authors replaced the Western Blots in Figures 2B and 2D in the submitted article with new Western Blots in Figure 3C to consolidate the results into one blot and add results for VEGF with GW9662, which had been missing in the submitted version. The authors say Figure 3C does match the text description in the article, is not their experimental result, and they do not know how it was included in the article.
